# Group-Specific Multiplex PCR Detection Systems for the Identification of Flying Insect Prey

**DOI:** 10.1371/journal.pone.0115501

**Published:** 2014-12-19

**Authors:** Daniela Sint, Bettina Niederklapfer, Ruediger Kaufmann, Michael Traugott

**Affiliations:** Institute of Ecology, University of Innsbruck, Innsbruck, Austria; Swedish University of Agricultural Sciences, Sweden

## Abstract

The applicability of species-specific primers to study feeding interactions is restricted to those ecosystems where the targeted prey species occur. Therefore, group-specific primer pairs, targeting higher taxonomic levels, are often desired to investigate interactions in a range of habitats that do not share the same species but the same groups of prey. Such primers are also valuable to study the diet of generalist predators when next generation sequencing approaches cannot be applied beneficially. Moreover, due to the large range of prey consumed by generalists, it is impossible to investigate the breadth of their diet with species-specific primers, even if multiplexing them. However, only few group-specific primers are available to date and important groups of prey such as flying insects have rarely been targeted. Our aim was to fill this gap and develop group-specific primers suitable to detect and identify the DNA of common taxa of flying insects. The primers were combined in two multiplex PCR systems, which allow a time- and cost-effective screening of samples for DNA of the dipteran subsection Calyptratae (including Anthomyiidae, Calliphoridae, Muscidae), other common dipteran families (Phoridae, Syrphidae, Bibionidae, Chironomidae, Sciaridae, Tipulidae), three orders of flying insects (Hymenoptera, Lepidoptera, Plecoptera) and coniferous aphids within the genus *Cinara*. The two PCR assays were highly specific and sensitive and their suitability to detect prey was confirmed by testing field-collected dietary samples from arthropods and vertebrates. The PCR assays presented here allow targeting prey at higher taxonomic levels such as family or order and therefore improve our ability to assess (trophic) interactions with flying insects in terrestrial and aquatic habitats.

## Introduction

Molecular methods have been widely adopted to investigate trophic interactions and proved to be especially useful to disentangle feeding relationships accurately and quickly [1], [2]. While initially, interactions between two or a few specific species were addressed [3]–[5], during the last years the focus moved from identifying those simple, linear relationships towards approaches characterizing food web interactions in whole communities [6]–[9]. As molecular diet analysis provides a snapshot picture of the dietary choice of individual consumers [2], the robustness of the trophic data generated depends on a high enough number of consumers screened for food DNA. Today, two molecular approaches, both of which have specific strengths and weaknesses, are generally used: (i) diagnostic PCR using taxon-specific primers where food identification is inferred from the presence/absence of prey-specific amplicons and (ii) sequence-based identification employing general primers for barcoding short stretches of food DNA using next generation sequencing (NGS) techniques [2]. The latter approach is increasingly used due to its appealing ability to read sequence information out of complex DNA mixtures and constantly decreasing costs. Next generation sequencing is well suited to provide detailed dietary information for individual consumers or for whole populations of consumers (i.e. when individual samples are pooled). The latter, however, is restricted by the need to label the dietary samples using individual multiplex identifier (MID) sequences which are attached to the barcoding primers along with platform-specific fusion primers (i.e., the MID identifier allows assigning the generated sequences to individual consumers). This restricts the analysis of large sample numbers as these labelled primers are costly. The elongation of PCR primers with those long non-binding sequences can also influence performance during PCR (i.e. potentially introduce methodologically-induced variability in the data set), requiring adaptation of the reaction conditions [10]. Although the latest developments may allow to circumvent the purchase of many different fusion primers in the future [10], the number of samples that can be pooled for a NGS run still is limited. This makes it necessary to conduct several sequencing runs to investigate many hundreds or thousands of samples individually.

When the consumer and its food are not closely related as for example in herbivores [11], [12], insectivorous mammals [13] or piscivorous birds and mammals [14], [15], highly conserved primers targeting only the food-DNA can be applied. This avoids another common problem of NGS-based approaches, the co-amplification of consumer-DNA which is usually present in high copy numbers compared to the prey DNA and which can significantly reduce the number of informative prey sequences [16]. In those situations, where the primer pair targeting the prey sequences will also amplify the consumer, blocking primers need to be developed which prohibit the amplification of consumer DNA. However, this might also block an unknown part of the prey DNA. This risk is especially high if closely related species might feed on each other (e.g. insectivorous insects or piscivorous fish). Besides this and other known problems involved in the development of blocking primers [17], their development will in practice only be manageable if few consumer species are investigated and in some cases it can be impossible to develop a full suite of consumer-specific blocking primers allowing to investigate the prey taken by a community of closely related predators.

The application of diagnostic PCR using taxon-specific primers, although restricted to the prey taxa targeted by the primers, can overcome the limitations inherent to the NGS approach. Hundreds to thousands of individual consumer samples from different taxa can be tested rapidly and at comparably low costs for a specific number of food sources. Over the last years taxon-specific primers were designed for a wide range of plant and animal food sources [1], [18], [19] which can easily be applied to track trophic links via diagnostic PCR. The ability to investigate whole species communities via taxon-specific primers was supported by the rapid progress made in multiplex PCR techniques [20]. In multiplex PCR systems several trophic links can be analysed within a single PCR as the individual targets result in amplicons of different length and can consequently be identified without additional sequencing steps [21]. Although there is an initial investment when developing these multiplex PCR systems, this is redeemed when large numbers of samples need to be tested, as they can be processed in a short time. Consequently, more and more multiplex PCR systems are developed and applied to answer all types of biological questions [20].

In molecular trophic ecology mostly species-specific primers have been applied in singleplex and multiplex PCR assays to investigate trophic links directly [1]. These primers are well suited to track interactions of certain species precisely. However, they do not permit to assess the wider dietary range of generalist predators due to the often large number of potential prey species within a specific taxon (e.g. a family or order). In that case, diagnostic primers targeting whole groups of organisms are needed which would allow examining the food spectrum on a more general level [22], [23]. Combined with species-specific primers, they could also be applied in a two-step approach to unravel complex feeding interactions in detail: first, the prey is assigned to a higher taxonomic level which can then be further resolved in a second step with more specific primers [24].

Although the application of group-specific primers is appealing, only few group-specific primers have been published so far to investigate feeding networks in terrestrial systems. This limits the possibilities to characterize trophic linkages on a higher taxonomic level using diagnostic PCR. Those group-specific primers which are available focus primarily on agricultural systems, targeting taxa such as collembolans [22], earthworms [25], dipterans [23], aphids [26], or different plant families [18]. Although insects are among the most important food sources worldwide (non-insect insectivores are reported from six phyla and 13 classes including Arachnida, Chilopoda and most classes of vertebrates [27]) only few group-specific primers for insects have been developed so far. This is especially true for common groups of flying insects such as Lepidoptera, Hymenoptera and different families within the Diptera. This shortage of group-specific primers is due to the difficulties involved in the design of such primers. The nature of a group-specific primer is that it amplifies DNA of a certain set of targeted taxa, but not of others. This means, primer binding sites have to be located in semi-conserved DNA regions, where all target taxa share the same (or at least a very similar) DNA sequence, but non-target DNA is different enough to prevent amplification [28], which can be difficult to achieve.

A common way to generate group-specific primers at a higher taxonomic level is to select DNA regions that only occur in the targeted group such as chloroplast DNA for the amplification of plants in animal consumers [29]. This approach, however, is not suited to design primers for different animal taxa, as the target and non-target groups are more closely related within a phylum than between and thus no such general differences in the genome are present. Another DNA region often targeted for molecular investigations is the cytochrome *c* oxidase subunit I (COI) gene, which was selected as the barcoding region for animals, as it provides sufficient variability to differentiate between species, although not equally well in all taxa [e.g. 25, 30]. Due to this high inter-specific variability the COI is suited to design species-specific primers, but this aspect precludes at the same time the development of primers targeting higher taxonomic levels such as families. The nuclear multicopy 18 s ribosomal DNA (rDNA) gene however, proved to be a good candidate to fulfil the requirements for semi-conserved DNA, i.e. to assemble groups of related taxa using shared primer binding sites.

The aim of this study was to remedy the deficit of group-specific primers and develop primers for various taxa of flying insects including the orders Lepidoptera, Hymenoptera, Plecoptera and several families of Diptera based on 18s rDNA sequences. The assemblage of the primers in multiplex PCRs allows simultaneous detection of these taxa. Initially, the primers were developed on the basis of flying insects occurring in high Alpine glacier forelands on pioneer successional stages. However, as these primers are targeting higher taxonomic levels, the assays can be applied beneficially also in other ecosystems for the identification of flying insect DNA. To prove their suitability in non-Alpine food webs, a wide range of target and non-target organisms of other habitats was tested empirically and *in silico* on DNA sequences available in public databases. Finally, the new group-specific multiplex PCR systems for flying insect prey were tested on field-collected samples of generalist arthropod predators and bat faeces to check for their general suitability to track the consumption of flying insects.

## Materials and Methods

### Sampling of animal reference material

A large collection of DNA extracts comprising many different animals from various habitats is available in our lab for developing and testing of molecular assays. This collection was supplemented with animals specifically collected for the development of the group-specific primers targeting flying insects. Flying insects were sampled during the snow-free period between 4^th^ July and 13^th^ August 2009 in the glacier foreland of the 'Rotmoosferner' (46.824 N, 11.045 E; Tyrol, Austria) at approx. 2400 m a.s.l. with malaise traps and grey and yellow bowls. The traps were emptied daily and all collected insects transferred to 96% ethanol. Predatory arthropods were collected alive within dry pitfall traps, starved for one week to allow digestion of prey remains and then freeze-killed. Additionally, linyphiid spiders were hand collected and freeze-killed after starvation. No specific permissions were required for the arthropod sampling within the glacier foreland and the sampling did not involve endangered or protected species.

### Assay development

Predators were identified to species level, dipterans to family level and all other arthropods were processed at order level. The DNA of several individuals per target-taxon included in the multiplex PCR systems (see below) and many non-target taxa like other dipteran families, beetles, spiders, harvestman, mayflies, caddisflies and collembolans were extracted with a silica-based kit (DNeasy blood and tissue kit; QIAGEN, Hilden, Germany). Part of the 18 s rDNA was amplified and sequenced using the primers 18sL0001 and 18sR1100 [31]. Each 10 µl reaction mix contained 1.5 µl DNA extract (typically 2–200 ng DNA), 5 µg bovine serum albumin (BSA), 0.5 µM of each primer, 0.2 mM dNTP (Genecraft, Köln, Germany), 1× reaction buffer (NEB, Ipswich, U.S.), 3 mM MgCl_2_ (NEB), 0.25 U oneTAQ (NEB), and PCR grade water to adjust the volume. Cycling conditions in an Eppendorf Mastercycler (Eppendorf, Hamburg, Germany) where 2 min at 94°C, 35 cycles of 20 s at 94°C, 30 s at 50°C, 90 s at 68°C and final elongation for 3 min at 68°C. Sequencing of PCR products was conducted by Eurofins MWG Operon (Munich, Germany). In addition to the primer pair stated above, the internal primer 18sR0532 [31] was used for sequencing.

Generated DNA sequences were processed and aligned using BioEdit [32] and primers were designed with Primer Premier 5 (PREMIER Biosoft International, Palo Alto, U.S.) targeting the orders Plecoptera, Lepidoptera, Hymenoptera, conifer aphids within the genus *Cinara* and abundant dipteran families including Anthomyiidae, Calliphoridae, Muscidae, Phoridae, Syrphidae, Bibionidae, Chironomidae, Sciaridae, and Tipulidae. A single primer pair amplifying several families of the subsection Calyptratae (Anthomyiidae, Calliphoridae, Muscidae, Scatophagidae, Tachinidae) was developed, as it was not possible to find primer binding sites distinguishing those families based on the targeted region of the 18s rDNA. The allochthonous coniferous aphids were identified morphologically by a specialist (E. Schliephake, Julius Kuehn Institute (JKI), Quedlinburg, Germany) and are very probably *Cinara cembrae*. However, they could not be identified to species level with certainty due to the lacking knowledge of the host plant and consequently we refrain to state that the developed primers are specific to *C. cembrae*, but use *Cinara* sp. instead. As the primers are targeting 18s rDNA, they are likely to amplify also DNA of related species, but neither sequence information nor DNA was available to test this assumption. The properties of the different primers and resulting DNA fragment lengths were selected to allow grouping of the primers into two multiplex PCR systems amplifying five (FLY-1) and six (FLY-2) taxa of flying insects, respectively.

To ensure that all multiplex PCR systems are well suited for molecular gut content analysis, the primers were designed to amplify DNA fragments no longer than 300 bp [19].

After testing of the individual primer pairs in singleplex reactions to check for correct amplification, optimal annealing temperatures were determined with gradient PCRs and primer concentrations were adjusted to balance amplification success for all fragments using standardised DNA templates as described in Sint *et al*. [20]. PCR reactions were based on the Type-it Mutation Detect PCR Kit (QIAGEN) with the addition of BSA to reduce potential inhibition if applied for gut content analysis [33]. In the multiplex PCR system FLY-1 tetramethylammonium chloride (TMAC) was added to enhance specificity [34]. PCR products were separated and analysed using QIAxcel, an automatic capillary electrophoresis system with the corresponding software QIAxcel BioCalculator (QIAGEN). DNA fragments resulting in a signal strength of ≥0.1 relative fluorescent units (RFU) were defined as positive detection.

The sensitivity of the optimised multiplex systems was determined with and without the addition of approx. 300 ng of non-target DNA (Lithobiidae) using standardised numbers of DNA templates. An extensive specificity testing comprising 381 individuals from 196 taxa of arthropods, gastropods and vertebrates inhabiting different ecosystems was conducted to check for correct amplification of target taxa and exclude false amplification of non-target organisms. A list of all empirically tested targets and non-targets with their corresponding amplification results is provided as S1 Material.

### *In silico* PCR

DNA sequences were downloaded from GenBank [35] and SILVA [36] based on the search terms Diptera, Hemiptera, Hymenoptera, Lepidoptera, and Plecoptera. The extension 18 s was used when searching GenBank to download only relevant data and RNA sequences from SILVA were translated into DNA. Based on these sequences *in silico* PCRs were performed with CLC Main Workbench 6 (CLC bio, Aarhus, Denmark) using the ‘Find Binding Sites and Create Fragments' tool. Detailed settings are reported in S1 Material.

### Testing of field-collected samples

To test the multiplex PCR systems on ‘real world samples’, arthropod predators were collected in an area ice-free for less than 20 years in the Alpine glacier foreland of the ‘Rotmoosferner' between 7^th^ and 11^th^ July 2010. Non-invasive regurgitates from predatory carabid beetles (*Nebria germari*, *N. jockischii*, *N. rufescens*, *Oreonebria castanea*) and whole individuals of linyphiid and lycosid spiders (*Pardosa nigra*, *P. saturatior*) were collected, freeze killed (spiders only) and stored at −28°C until DNA extraction. Unlike the beetles, spiders could not be identified easily to species level upon collection, and were consequently handled at genus (*Pardosa*) and family level (Linyphiidae).

DNA extraction followed a modified CTAB protocol [37] and two extraction negative controls were included in each batch of 30 samples to check for carry-over contamination. Due to the considerable size of the lycosid spiders, their DNA extracts still contained many potentially inhibiting substances after the CTAB extraction and they were additionally cleaned with the QIAquick PCR Purification Kit (Qiagen). Total DNA concentration of the different sample types was measured from 10 randomly chosen samples per species with a NanoDrop ND-1000 (Thermo Scientific, Waltham, USA) and ranged from <1–10 ng/µl in regurgitates, 2–50 ng/µl in Linyphiidae and 40–150 ng/µl in *Pardosa*.

A total of 272 arthropod samples (52 *N. germari*, 45 *N. jockischii*, 30 *N. rufescens*, 70 *O. castanea*, 26 *Pardosa* spp., 49 Linyphiidae) were screened with the two newly developed multiplex PCR systems to test the assays' suitability for diet analysis.

Eleven faecal pellets of the greater horseshoe bat (*Rhinolophus ferrumequinum*), were collected during summer 2013 in a colony inhabiting the church of Schluderns (South Tyrol, Italy) and preserved separately in 96% ethanol. Before DNA extraction with the DNeasy blood and tissue kit (QIAGEN), the ethanol was allowed to completely evaporate in a digestor. All samples were then tested with both newly developed multiplex PCR systems.

Field-collected samples where no DNA could be amplified with the multiplex PCR systems were tested for the presence of amplifiable DNA with either a multiplex PCR system screening for DNA of the predators and collembolans [38] (beetle regurgitates and *Pardosa* spp.) or with singleplex PCRs using general primers [LCO1490/HCO2198, 39] (linyphiid spiders, where un-degraded DNA of the consumer is present). Samples where no DNA could be amplified (four beetle regurgitates and three linyphiid spiders) were excluded. During screening all extraction negative controls were tested as if they were samples and two PCR positive controls (artificial mix of all templates) and six PCR negative controls (water instead of DNA template) were included per 96 well plate in each PCR to check for correct amplification and DNA carry-over contamination.

## Results

### Multiplex PCR assays

Two multiplex PCR systems were developed amplifying five and six abundant taxa of flying insects, respectively. The first multiplex PCR system FLY-1 amplifies DNA from members of the Calyptratae, Phoridae, Plecoptera, Sciaridae, and Tipulidae, whereas the second system FLY-2 targets Bibionidae, Chironomidae, *Cinara* sp., Hymenoptera, Lepidoptera, and Syrphidae. The primer sequences, the corresponding fragment length of the different amplicons and the primer concentrations in PCR are given in Table 1. All reactions were performed using the Type-it Mutation Detect PCR Kit (QIAGEN) in a volume of 10 µl. Each reaction contained 1.5 µl DNA extract, each primer at its corresponding concentration (Table 1), 1× Type-it reaction mix, 5 µg BSA, 0.25× Q-Solution for FLY-1 only, 60 mM TMAC for FLY-1 only and PCR grade water to adjust the volume. The optimised cycling conditions on an Eppendorf Mastercycler included an initial activation step of 5 min at 95°C, 35 cycles of 30 s at 95°C, 3 min at 60°C (FLY-1) or 66°C (FLY-2), 1 min at 72°C and final elongation for 10 min at 68°C.

**Table 1:** Primer pairs included in the multiplex PCR systems FLY-1 and FLY-2.

Multiplex	Target	Primer	Primer sequence (5'-3')	size (bp)	conc. (µM)
**FLY-1**	Phoridae	Pho-gen-S265	TTCTTTCGGGGATCGTTGAC	82	0.2
		Pho-gen-A266	GACATTTGAAAGATCTGTCGTCG		
	Plecoptera	Ple-gen-S268	TATGGTTCCTTAGATAATACACCA	117	0.2
		Ple-gen-A269	GGTTTTGATCTAATAAAAGCGT	(117–118)	
	Tipulidae	Tip-gen-S267	GCATGTCTAAGTACACACTCTCG	159	0.1
		Tip-gen-A268	ATAAAAGCACACGTTCCTTG		
	Sciaridae	Sci-gen-S266	AGAAACCGGTAAAATGGGT	187	0.2
		Sci-gen-A267	AACCAAGGTAATCCAAGACAT	(186–188)	
	Calyptratae	Cal-gen-S263	AAAATAACAATACAGGACTCATATTA	238	0.3
		Cal-gen-A264	TAATACGCTTACATACATAAGGTATA	(236–240)	
**FLY-2**	Syrphidae	Syr-gen-S269	ATTAGGCTAAAACCAAGCGATTT	86	0.8
		Syr-gen-A270	TCGGTACAAGACCATACGATCG		
	Hymenoptera	Hym-gen-S273	CGATGTTGGTTCACCGCTC	114	0.2
		Hym-gen-A274	CRATGAAGAGCACCGCGAT	(101–200)	
	Lepidoptera	Lep-gen-S274	GCAAGCCGTATTAAGGCGAT	134	0.3
		Lep-gen-A275	CCCATCGCTGGTCAGAGTTC	(133–135)	
	Bibionidae	Bib-gen-S271	TTCCGCACAGGCAATACCTT	153	0.2
		Bib-gen-A271	CAATAAAGAGAACTGCTATGGGCT		
	Chironomidae	Chi-gen-S272	CCGTCAAAAGTTTCTTGTGCAG	179	0.2
		Chi-gen-A272	CGTAGCAACCATGGTAGTCCTCT	(175–188)	
	*Cinara* sp.	Cin-sp-S275	TCTGGGCGGTGTCGGAC	251	0.08
		Cin-sp-A277	GCACAGCAAGATTGGAGTAGG		

Columns show the targets and corresponding primer names (A and S denote forward and reverse primers, respectively), primer sequences in 5′-3′ direction, the size of the resulting amplicon (if the *in silico* PCR reported variable amplicon lengths, the most frequent value is given with the full range in parenthesis) and the final concentration of each primer in the multiplex reaction.

**Figure 1:**
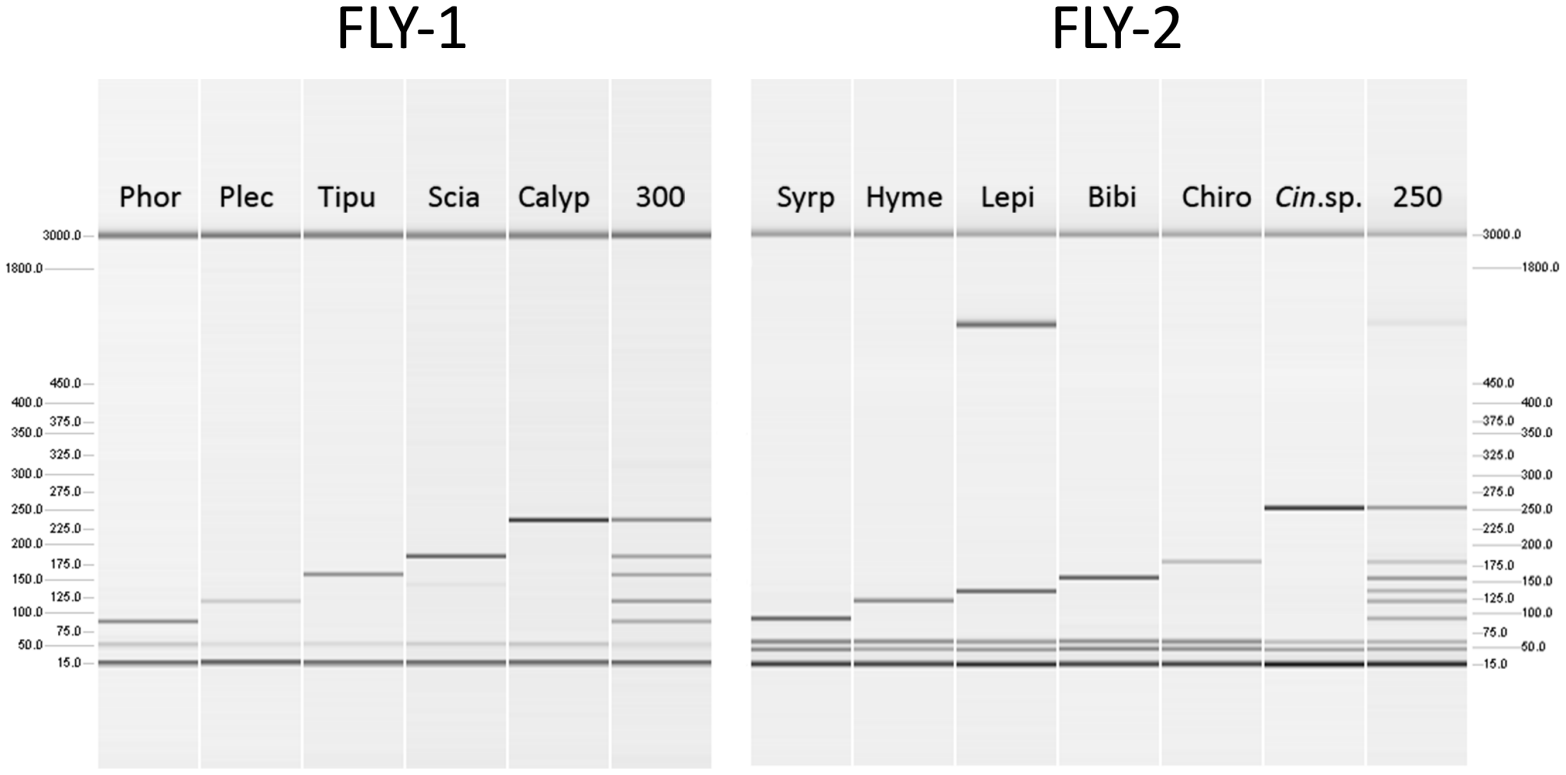
Gel image of PCR products amplified with the multiplex PCR systems FLY-1 and FLY-2 and separated with QIAxcel. FLY-1: Phoridae (Phor), Plecoptera (Plec), Tipulidae (Tipu), Sciaridae (Scia), Calyptratae (Calyp), artificial mix containing 300 double stranded (ds) templates per target. FLY-2: Syrphidae (Syrp), Hymenoptera (Hyme), Lepidoptera (Lepi), Bibionidae (Bibi), Chironomidae (Chiro), *Cinara* sp. (Cin.sp.), artificial mix with 250 ds templates per target. Note that an internal marker is run alongside each sample (15 and 3000 bp) and the scale on the left and right side, respectively, allows an estimation of fragment length. Sciaride may result in an additional weaker amplicon of ∼140 bp which is not interfering with another fragment in the system; the same applies for the long fragment that can be amplified from lepidopterans.

Signal strength of the different amplicons was balanced within each multiplex PCR system when tested with standardized PCR templates [38] and the fragments could be easily separated from each other with QIAxcel (Fig. 1). Both systems proved to be sensitive and 60 double stranded template molecules were sufficient to amplify a well detectable PCR product (i.e. signal strength ≥0.1 RFU on QIAxcel) with FLY-1. The sensitivity of FLY-2 was even higher as less than 20 template molecules produced a product ≥0.1 RFU. The presence of a large amount (∼300 ng) of non-target DNA did not alter the observed sensitivity in FLY-1. The sensitivity of three targets in FLY-2 was reduced to some extent, but still 60 template molecules were sufficient for the detection of Lepidoptera and Bibionidae and 120 molecules for the detection of Chironomidae. Note, that in FLY-1 samples from Sciaridae might amplify a weak double band at ∼140 bp length and in FLY-2 lepidopteran specimens can produce an additional fragment of about 800 bp length (Fig. 1). As the longest target fragment included in FLY-2 is 251 bp for *Cinara* sp., this extra band will not interfere with the diagnostic amplicons. The same is true for FLY-1, where no diagnostic fragment is generated at 140 bp.

### Specificity testing and *in silico* PCR

The performed *in silico* PCRs and empirical target testing demonstrated that the primers are suitable to amplify DNA from a broad range of species within the respective taxa which occur in various ecosystems. Target DNA was successfully amplified from all empirically tested Lepidoptera (25 indiv.), Plecoptera (5), Bibionidae (6), Chironomidae (13), Phoridae (4), Sciaridae (7), Syrphidae (27), and Tipulidae (4). Most individuals belonging to the calyptrate families Anthomyiidae (8), Calliphoridae (8 of 9), Muscidae (14), Scatophagidae (3 of 4), and Tachinidae (6 of 8) could be amplified as well. The tested taxa included many common species such as important lepidopteran pests, 17 species of central European syrphids and various parasitic and non-parasitic hymenopterans (see S1 Material). Hymenoptera was the only group of flying insects, where a significant number (25 out of 69) of the empirically tested individuals did not amplify the expected DNA fragment. To evaluate if amplification success increases when the annealing temperature is lowered, the hymenopteran samples were tested in the FLY-2 multiplex PCR assay at 62°C. This reduction of the annealing temperature increased the number of individuals from which DNA could be amplified with the hymenopteran-specific primers to 55, leaving only few taxa (e.g. *Aphidius* spp.) that could still not be detected (S1 Material). The analysis of the results from the *in silico* PCR revealed that out of 2,458 hymenopteran sequences covering both primer binding sites downloaded from GenBank, 1,881 produced a fragment with the developed hymenopteran primers. Only two families were accounting for 82% of all 'failed amplifications': Braconidae and Formicidae where 54 sequences out of 221 and 417 out of 520, respectively, did not produce a fragment. For the remaining 81 hymenopteran families present in this set of sequences, the taxonomic coverage, referred from *in silico* PCR, was 93.8%.

The *in silico* PCR indicated that the primers targeting different families of the Calyptratae (Anthomyiidae, Calliphoridae, Muscidae, Scatophagidae, Tachinidae) might as well amplify DNA of the other families belonging to this subsection of the Diptera (S2 Material). For most taxa the variation in the fragment length generated via *in silico* PCR was a maximum of ±2 bp of the most frequently reported length. The variation was slightly higher in Chironomidae (175–188 bp) but due to the sufficient distance to neighbouring fragments in the multiplex PCR, this will not cause difficulties in identifying the amplicon. The highest variability was found in Hymenoptera (S2 Material): from the 1,929 fragments generated based on the GenBank sequences, 84.2% had a length between 110 and 120 bp and can thus be well differentiated from the neighbouring fragments of Syrphidae (86 bp) and Lepidoptera (133–135 bp). Another 12% of the hymenopteran fragments ranged between 101 and 109 bp (5 fragments) or between 121 and 128 bp (227 fragments). Only 72 individuals (3.7%; many of them parasitic scelionid wasps) showed a fragment length >128 bp which would overlap in fragment size with the PCR product generated from other targeted DNA

### Application of the multiplex PCR systems to field samples

The two multiplex PCR systems turned out to be well suited for diet analysis in arthropod predators. All targeted taxa where detected in the field-collected samples (Table 2), except for the brachyceran family Phoridae. In 19% of all tested arthropod predators DNA of at least one prey taxon was detected with a maximum of four different prey types amplified from a single lycosid spider.

**Table 2:** Detection of flying insect prey DNA within dietary samples of different consumers (Linyphiidae, *Pardosa* spp.: whole body samples; *Nebria germari*, *N. jockischii*, *N. rufescens*, *Oreonebria castanea*: regurgitates; *Rhinolophus ferrumequinum*: faecal samples) when tested with the newly developed multiplex PCR systems FLY-1 and FLY-2.

		Linyphiidae	*N. germari*	*N. jockischii*	*N. rufescens*	*O. castanea*	*Pardosa* spp.	*R. ferrumequinum*
	n	46	52	45	29	67	26	11
**FLY-1**	Calyptratae	2	0	0	0	0	2	0
	Phoridae	0	0	0	0	0	0	0
	Plecoptera	0	0	1	0	0	0	0
	Sciaridae	0	2	1	0	1	2	0
	Tipulidae	0	0	1	4	2	2	7
**FLY-2**	Bibionidae	0	3	0	0	0	0	0
	Chironomidae	0	1	3	0	0	0	0
	*Cinara* sp.	0	11	12	0	3	1	0
	Hymenoptera	0	0	2	0	1	3	5
	Lepidoptera	0	1	0	0	0	0	11
	Syrphidae	0	0	0	1	0	3	1

See main text for details on the multiplex PCR systems.

The percentage of individuals testing positive for DNA of flying insects was lowest in linyphiid spiders (4%). From the lycosid spiders 23% had consumed flying insects and within the carabid beetles the detection rate ranged between 9% (O. *castanea*) and 38% (*N. jockischii*) (Table 2). The aphid genus *Cinara* was the taxon of flying insects that was detected most frequently in the investigated arthropod predators and accounted for 41% of the detections.

DNA of flying insect prey could be amplified from all 11 faecal samples investigated of the greater horseshoe bat with the number of prey groups ranging in individual samples between one and three. All samples contained lepidopteran-DNA and in most cases also DNA of Hymenoptera and/or Tipulidae was detected (Table 2). Due to the observed variability in the fragment length of hymenopteran DNA, all faecal samples were re-screened in singleplex reactions with the hymenopteran and lepidopteran specific primers respectively, to check if any longer hymenopteran fragments could have been misinterpreted as Lepidoptera. The singleplex reactions resulted in exactly the same result as the multiplex PCR testing.

## Discussion

The two new multiplex PCR systems including group-specific primers for a variety of flying insect taxa were highly sensitive and amplified all targets at approximately the same efficiency, resulting in an even signal strength for all amplicons.

While the applicability of multiplex PCR systems including species-specific primers is *per se* limited to ecosystems where those species occur, the newly developed multiplex PCR systems targeting various flying insects at higher taxonomic levels can be used to track consumption of common dipteran families and abundant orders of flying insects in many different habitats. Until now, only few group-specific primers were available for diagnostic PCR to study trophic interactions of terrestrial invertebrates [22], [23], [25]. They were mainly designed to detect consumption of alternative prey by biological control agents and thus many species rich taxa occurring in terrestrial ecosystems, especially flying insects, were not covered. Beside several primers targeting aquatic macroinvertebrates, where some orders have winged imagines [40], [41] and a dipteran primer developed by King *et al.* [23], no general primers for flying insects were available up to now. The dipteran primer pair developed by King et al. [23], although amplifying several dipteran taxa, will not amplify two important nematoceran families, i.e. Chironomidae and Tipulidae and is thus restricted in examining consumption of dipteran prey.

The newly developed multiplex PCR systems presented here allow a general investigation of predation on flying insects at order level and a more detailed analysis of consumption of different dipteran families. With this level of resolution, they are general enough to be applicable to many different predator-prey systems in terrestrial as well as in aquatic habitats. The principal applicability of the multiplex PCR systems in various habitats was confirmed by testing them empirically with faecal samples of bats and a wide range of target and non-target organisms from various aquatic and terrestrial habitats, including vertebrates. No false amplification of non-target organisms was observed. Only the primer pairs targeting Hymenoptera and the dipteran subsection Calyptratae did not amplify with all empirically tested target individuals and in the *in silico* PCR (S1 and S2 Material). Still, the amplification success of these primers was well above the taxonomic coverage reached by many primer pairs deemed 'general' [42]. With more than 100,000 described species, the Hymenoptera are amongst the most diverse animal orders [43]. Hence, it is not surprising that some species (mainly from the Braconidae and Formicidae) were not amplified with the newly developed order-specific primer pair for Hymenoptera as their sequences are too variable at the primer binding sites for successful annealing in every species. Still, the majority of the hymenopteran species tested resulted in a successful amplification of the target fragment. By reducing the annealing temperature for the FLY-2 assay to 62°C it was possible to nearly halve the number of hymenopteran samples not testing positive. If the primer pair is applied in a singleplex reaction, one could lower the annealing temperature even further to enhance the amplification of targets with imperfect primer match. However, this increases also the probability that non-target amplification occurs. A specificity testing on a set of 94 non-target organisms covering all major taxa included in the full specificity testing of the multiplex PCR systems was performed under the same conditions as for FLY-2 with the exception that only the primer pair amplifying hymenopteran DNA was included and the annealing temperature was lowered to 62°C. This singleplex test did not reveal any amplification of non-target DNA, suggesting, that the primers amplifying hymenopteran DNA can be applied under these more relaxed conditions. Still, we do not suggest to apply the full multiplex PCR system at an annealing temperature below 66°C (without carefully checking the specificity in the respective study system), as the chance of unspecific DNA amplification by any two of the many primers included in the multiplex PCR system is high.

An *in silico* PCR, albeit delivering valuable information, cannot fully replace an empirical target/non-target testing [44]. Many more factors influence the outcome of a PCR than can be adjusted and specified in a computer programme checking sequence similarity, so that the empirical and *in silico* results can diverge from each other. Especially the DNA polymerase employed, but also additives and thermocycling conditions strongly influence PCR specificity and efficiency [45], and consequently amplification success, factors which are usually not accounted for in computer programmes simulating PCR. One example for such a difference between *in silico* and *in vitro* PCR was found for the primers of *Cinara* sp.: while the *in silico* PCR produced an amplicon for *Acyrthosiphon pisum* (S2 Material), no DNA fragment was amplified *in vitro* (S1 Material). Similarly the *in silico* PCR suggests, that the developed primers for Calyptratae will amplify the DNA of additional families in this subsection of the Brachycera. However, no amplicon was generated *in vitro* from the family of Sarcophagidae, where three individuals were tested empirically with the multiplex system (S1 Material). For the other families of this subsection, the amplification could not be verified/falsified *in vitro*, as no individuals from those families were available for DNA extraction.

In general, the observed fragment length for the different taxa of flying insects was stable. Only in the hymenopterans significant variation in the size of the PCR product was observed. However, in this order the majority of the species tested also gave a fragment length that could be clearly separated from other diagnostic fragments, making this primer pair a valuable tool for trophic analysis and other diagnostic applications where hymenopteran DNA needs to be detected and identified. The results of the *in silico* PCR indicated that some of the variation in the amplicon length in Hymenoptera is relatively stable within certain families (i.e. Braconidae, Eucharitidae). However, the converse argument, that a specific amplicon length points towards a specific hymenopteran family, will not hold true as variable amplicon lengths were also observed within other hymenopteran families, although rarely. This pattern of varying amplicon size can be an important consideration if primarily individuals of those families, generating amplicons with a length close to the next longer fragment in the multiplex system (Lepidoptera 133–135 bp), are expected to be detected in field samples. In such cases, attention should be paid to the separation of the generated PCR products and the usage of a high resolution method is advisable to be able to separate the amplicons of the different targets. The potential error caused by the small percentage of hymenopteran individuals producing amplicons of elevated length with the here developed primers can be further reduced by re-testing ambiguous samples in a singleplex PCR. Consequently, the conclusions drawn from diagnostic results of trophic interactions generated by the application of the present multiplex PCR systems will be generally correct.

As an alternative to diagnostic PCR with group-specific primers, dietary DNA could be amplified with universal primers followed by cloning of PCR products or DNA sequencing or by a NGS approach to assess the food spectrum at a broader level [46]. However, this sequence-based prey identification can suffer from an incomplete coverage of the prey spectrum due to unsuitable primers or co-amplification of the consumer or both [46], [47]. Consequently, specific measures (e.g. blocking primers) have to be developed and applied to prevent consumer amplification and generate meaningful results with barcoding based methods [17]. When Pinol *et al*. [16] tested an approach to apply NGS on linyphiid spiders without any measure to reduce the amplification of the spider DNA, prey detection was merely partly successful. Only nematodes and collembolans, the most common prey, were reliably detected and the results were not reproducible as most prey types were only present in one of four individual sequencing runs. In general, the identification power of these sequence-based approaches depends to a large extent on the availability of good reference sequence databases, which are often lacking. In practice, this can lead to an unsatisfactory low resolution of the investigated food spectrum [e.g. 13, 48, 49]. Even misidentifications cannot be ruled out as lately shown by Clarke *et al.* [10], where COI sequences of Hymenoptera and Orthoptera were misidentified as Lepidoptera by a BLAST search.

The application of the here presented group-specific primers and the multiplex PCR systems is not restricted to investigate consumption of the target taxa, but the primers can be useful whenever any developmental stage of a targeted taxon has to be identified via its DNA. The primers could, for example, be applied to classify dipteran larvae and with these highly sensitive PCR assays, even non-destructive DNA extraction methods could be used. Another potential application is tracking of endoparasitism by tachinid flies and parasitic hymenopterans. In this case, however, the correct amplification of the parasitoid taxa of interest should be verified before investigating field samples.

In the present study, the analysis of the field-derived samples with the newly developed multiplex PCR systems mainly served to prove their applicability to ‘real world samples’. Therefore, we will refrain from a detailed interpretation and discussion of the outcomes of the gut content- and faecal analysis, as the limited number of tested samples will not allow drawing sound ecological conclusions but rather reflect a random snapshot.

Overall, the two new group-specific multiplex PCR systems presented here provide a quick and easy way to molecularly identify these taxa and to investigate the role of flying insects in the diet of insectivorous animals including arthropods, mammals, birds, reptiles, amphibians, and fish, helping to achieve a better understanding of their feeding ecologies.

## Supporting Information

S1 Material **List of all taxa included in the specificity testing of the newly developed multiplex PCR systems.**
(XLSX)

S2 Material **Settings and results of the** ***in silico*** **PCRs.**
(XLSX)
